# The Association between Mediterranean Diet and the Risk of Falls and Physical Function Indices in Older Type 2 Diabetic People Varies by Age

**DOI:** 10.3390/nu10060767

**Published:** 2018-06-14

**Authors:** Sigal Tepper, Amit Alter Sivashensky, Danit Rivkah Shahar, Diklah Geva, Tali Cukierman-Yaffe

**Affiliations:** 1 Department of Nutritional Sciences, Tel Hai Academic College, Upper Galilee 1220800, Israel; 2 Department of Epidemiology and Preventive Medicine, School of Public Health and Preventive Medicine, Sackler Faculty of Medicine, Tel Aviv University, Tel Aviv 69978, Israel; alteramit@gmail.com (A.A.S.); tcukierm@gmail.com (T.C.-Y.); 3 The S. Daniel Abraham Center for Health and Nutrition, Department of Public Health, Faculty of Health Sciences, Ben-Gurion University of the Negev, Beer-Sheva 8443944, Israel; dshahar@bgu.ac.il; 4 Department of Public Health, Faculty of Health Sciences, Ben-Gurion University of the Negev, Beer-Sheva 8443944, Israel; diklah.geva@gmail.com; 5 The Center for Successful Aging with Diabetes, Endocrinology Institute, Sheba Medical Center, Ramat Gan 52621, Israel

**Keywords:** elderly, type 2 diabetes, mediterranean diet, physical function

## Abstract

Background and Aims: Diabetes and dysglycemia increase the risk of frailty and decreased physical abilities. Adherence to the Mediterranean Diet (MD) may reduce this risk. We hypothesized that adherence to the MD is associated with physical function in older type-2 diabetic patients and that the association is stratified by age. Methods and Results: We recruited type-2 diabetes patients aged >60 years at the Center for Successful Aging with Diabetes at Sheba Medical Center. Health status and demographic data were obtained from medical records. Food Frequency Questionnaire was used for nutritional assessment and calculation of MD score. Physical function indices were determined by a physiotherapist and included: Berg Balance test, Timed Get-Up-and-Go, 6-min walk (6 MW), 10-m walk (10 MW), Four Square Step Test, 30-s chair stand and Grip strength, and activities and instrumental activities of daily living. Among 117 participants (age 70.6 ± 6.5), high adherence to MD was associated with better score on functional tests (low vs. high MD adherence: 9.7% vs. 25%, ANOVA *p* = 0.02). A significant age by MD interaction was found: a higher adherence to MD was associated with a better 6 MW (low vs. high: 387 ± 35 m vs. 483 ± 26 m; *p* = 0.001) and higher 10 MW (low vs. high: 1.8 ± 0.16 m/s vs. 2.0 ± 0.13 m/s; *p* = 0.02) in participants aged >75 years. These associations remained significant after controlling for gender, age, BMI, and physical activity. Conclusion: In the current study, we showed relationships between strength, physical performance, and MD among older diabetic patients. Future studies are needed to confirm this association and establish temporal relationships.

## 1. Introduction

Evidence from the last decade has shown that diabetes and dysglycemia are risk factors for unhealthy aging, including cognitive dysfunction [1], dementia [2,3], depression [4], and disability [5]. People with diabetes are less physically active and find simple and complex activities of daily living more difficult than those without diabetes after controlling for age [6]. People with diabetes also have a higher risk for falls and fractures [5,7]. Recent data suggests that this increased risk is due not only to recognized diabetes complications [8], but also due to an accelerated decline in physical capacity due to lower muscle quality and a more rapid decline in muscle mass and lower extremity strength over time [9,10].

The number of older people over the age of 60 with diabetes is increasing: 30% of the population over the age of 60 in the United States have diabetes. According to the Israel National Quality Program, 29% of the population age 65–74 and 33% of those over the age of 75–84 have diabetes [11]. Diabetes is associated with high risk for disability, and accelerated rate of decline in physical capacity [12]. Thus, elucidating protective modifiable factors becomes a priority. One such factor is a healthy diet, particularly the Mediterranean Diet (MD) [13,14,15].

The MD is characterized by a high intake of vegetables, fruits, legumes, and cereals; a moderate to high intake of fish; moderate to low consumption of poultry, meat, and dairy; high intake of monounsaturated fatty acid (mainly from olive oil); and a moderate amount (1–2 portions) of wine. Higher adherence to an MD has been shown to be associated with a decrease in number of cardiometabolic disorders [16], frailty, and disability, and improved physical function [13,17,18,19].

Several studies have examined the relationship between adherence to MD and physical capacity as measured by various indices. In a study performed on data from HealthABC [13], walking speed as a measure for mobility performance over 8 years was faster in older people with higher MD adherence at baseline. Findings in the same direction were shown in an analysis of the InChianti study on mobility decline [17] as well as in the ILSA study [18]. In a cross-sectional study performed by our group using data from nutrition and health surveys in Israel and in the United States, ADL (activities of daily living) measurements in Israel and 20-foot walk and strength of the knee extensors were both related to adherence to MD [19].

Less is known regarding this relationship in older people with diabetes. We identified only one recent publication from the Nurses’ Health Study [7] showing that the score for adherence to the alternate Mediterranean diet (aMED) (mainly fruits and vegetables) was related to frailty occurrence. In this study the MD score was modified to go along with the American diet, as an example of a non-Mediterranean country. Thus, the current study will test the hypothesis that a higher adherence to an MD in a Mediterranean country is associated with walking speed, better scores on indices of physical function, and better scores on tests that assess the risk for falls in this high-risk population. It will also examine if these relationships differ by age.

## 2. Methods

### 2.1. Study Population and Study Design

This is a cross-sectional study. Data regarding a convenience sample of the first 117 consecutive individuals with diabetes over the age of 60 who attended an evaluation day at the Center for Successful Aging with Diabetes at the Sheba Medical Center is included. The United Nations’ viewpoint that people aged 60+ are part of an older population was used for this pilot, as the aim of this study is elucidating risk and protective factors aiming at prevention in a relatively “healthy” population [20]. Included in the study were individuals with a diagnosis of type 2 diabetes, over the age of 60, with Hebrew native language. Excluded were individuals with significant hearing or visual disability, diagnosed dementia or cognitive impairment that in the view of the primary care physician may impair their ability to sign a consent form, any major non-diabetes related illness that may reduce life expectancy substantially or interfere with study participation, renal failure with a serum creatinine level above 2.0 mg/dL or significant liver disease, illiteracy, or a known history of stroke accompanied by disability that may impede their ability to perform the tasks required during the assessment. The study was approved by the Helsinki Committee of Sheba Medical Center as part of a study conducted at the Center for Successful Aging with Diabetes. All participants of the study signed an informed consent and participated in a comprehensive multi-disciplinary one-day evaluation at the Center.

### 2.2. Measurements

Demographic and health status data, physical examination, and lab values of diabetes status, diabetes duration, co-morbidities, and diabetes complications were obtained by history, physical examination, and blood work all participants were required to conduct prior to attending the evaluation day. Blood work that was within one month of the evaluation was accepted for this purpose.

### 2.3. Dietary Assessment

Dietary Assessment and calculation of Mediterranean Diet was performed by Food Frequency Questionnaire (FFQ) that was developed for the Israeli population [21] and validated for use in the older adults [22]. The development and validation process of the FFQ was described in detail elsewhere [21,22,23,24]. The FFQ includes 126 food items and presents 9 frequency options from “never or less than once a month” to “6 or more a day”. It is a semi-quantitative questionnaire with a standard portion size provided for each food item. The portion size estimates are based on information from the Israel Ministry of Health. The participants were asked about their average frequency of consumption during the past year. The questionnaire was self-administered electronically with the aid of an interviewer, thus ensuring completeness of the data as the participant is prompted when a question was not answered.

### 2.4. Mediterranean Diet Score

Adherence to the Mediterranean Diet was determined according to a score created in a previous study [19] and range from 0 to 9. For each of the nine components, with the exception of alcohol, a value of 0 or 1 was assigned. The units of measurements were serving size and the sex-specific medians of intake of the sample were used as cut-off points. One point was assigned to consumption over the median for each of the six protective components (fatty acid ratio, legumes, grains, fruits, vegetables, and fish). Participants received 1 point if their intake was below the median for the two non-protective components (dairy products and meat). For alcohol, if mean consumption was 10 to 50 g/day for men or 5–25 g/day for women 1 point was assigned. A score of 9 reflected maximum adherence by participants who meet all of the characteristics of the MD. Based on a sensitivity analysis we constructed 3 levels of adherence scores. Low adherence was defined as 0–2 points, medium adherence 3–4 points, and high adherence 5–9 points [19].

### 2.5. Physical Assessment

Assessment of physical capacity was conducted by a physiotherapist. This included the Timed-Get-Up-and-Go [25,26,27,28], 6-m walk, 10-m walk [29], Berg Balance Scale (BBS) [30], Four Square Step Test (FSST) [31], 30-s chair stand [32], and grip and pinch strength using a Jamar dynamometer [33,34]. In addition, an occupational therapist collected data pertaining to daily living activities and social engagement utilizing the Functional Independence Measure and the Frenchay activity index [35,36]. All data was collected, coded, and unified into a common database. The score on the Berg balance test was categorized using well-accepted cut-offs according to the risk for falls. Thus, scores below 36 indicate an increased risk for falls; scores between 37 and 45 indicate need of a walking aid in order to walk in a safe manner; scores > 45 indicate no increased risk of falls (independent walker).

File S1 (Supplementary Materials) presents a detailed description of the physical indices collected as part of the evaluation day procedure.

### 2.6. Statistical Analysis

We calculated the required sample size for multiple regression with ten predictors and medium effect size of F^2^ = 0.15, power of 1 – β = 80%, significance level of α = 0.05. This calculation using Soper’s [37] sample size calculator resulted with *n* = 118. This is an observational study and multiple testing was not accounted for.

Categorical variables were summarized as numbers and percent, continuous variables as mean ± SD. The distribution of all variables across adherence to MD categories was presented. Comparison between the distributions of the variables across MD categories was conducted by Chi square test for categorical variables, ANOVA for continuous variables, and Kruskal-Wallis tests for non-normally distributed variables.

Based on the age distribution and geriatric considerations, age was grouped into ≤75 years and >75 years. To further study the impact of MD on 6 MW and 10 MW, a generalized linear model (GLM) was fitted that included adherence to MD, age (up to and above 75), MD by age interaction as well as gender, BMI, and physical activity. Stepwise method was used to select the final cofactors in the GLM models. We considered the following variables in the initial multiple regression models: age, LDL, grip strength, Berg score, 6 MW, 10 MW, and Timed-Get-Up-and-Go. Afterwards, an intermittent model was examined that included all factors with significance level ≤0.25. We noted that MD also remained significant in the intermediate model. The reported model included only covariates significant at *p* < 0.05 for at least one subgroup/category.

The MD by age interaction term in the model allows testing the different effects of MD for significant covariates in regression models. Covariates included age, LDL, grip strength, Berg score, 6 MW, 10 MW, and Timed-Get-Up-and-Go for each age group, and the fitted values are graphically presented. All statistical analyses were performed using SPSS Statistics for Windows, Version 23.0 (IBM, Armonk, NY, USA).

## 3. Results

Data for the first consecutive 117 participants during the multi-disciplinary evaluation day is presented. Mean age was 70.6 (±6.5) with a mean of 15.6 (±2.7) years of education; 40% of participants were female, 76% were married, and 14.7% reported living alone (Table 1). Mean duration of diabetes was 16.7 (±10.3) years, 33.6% were insulin users with a mean HbA1c of 7.38% (±1.13), 34.5% had a history of IHD, 14.7% a history of CVD, 2.6% were current smokers, 97% reported a diagnosis of hypertension or took regular hypertension medications, and 97% had dyslipidemia.

### 3.1. Distribution of Variables across MD Category

There were no significant differences in the demographic and medical characteristics according to adherence to MD categories, as shown in Table 1.

Table 2 presents the physical, aerobic, strength, and balance scores. As shown, 11% of participants were found to be pre-frail, 3% frail, and 9.4% in need of a walking aid for safe walking. There was a significant difference in standardized grip strength score in the higher vs. lower adherence groups (low vs. high adherence −0.93 ± 0.82 vs.−0.29 ± 0.84; *p* = 0.03). High adherence to MD was related to being categorized in the “lower risk for falls” category according to the Berg Balance inventory: 9.7% vs. 25% in the highest compared with the lowest adherence to MD category (*p* = 0.02). Albeit not statistically significant, but noteworthy, are the differences in frailty and pre-frailty rates in the different categories with rates of 4.2% and 20.8%, respectively, in the low adherence to MD category versus 0% and 6.5%, respectively, in the high adherence to MD category (*p* = 0.2115, 0.5568, respectively). There was a difference (close to significance) in the number of meters walked in 6 min by those in the low adherence category (409 ± 143.5) vs. those in the highest category (477 ± 95.4) (*p* = 0.0658).

In regard to ADL and IADL (instrumental activities of daily living), those in the higher adherence to the MD category had a higher score on a scale that measures participation in complex activities of daily living including leisure activities and social engagement (Frenchay activity scale) (lowest vs. highest category 2.44 ± 0.5 vs. 2.79 ± 0.42; *p* = 0.0046).

### 3.2. Diet Quality across MD Categories

High MD adherence was associated with higher total energy and higher carbohydrate consumption, higher dietary fibers, iron, magnesium, potassium, sodium, selenium, vitamin E, vitamin C, vitamin K, folate, and vitamin B1, as well as higher consumption of fatty acids: monounsaturated, polyunsaturated *n*-3 fatty acid, and Trans fatty acids (Table S1 in the Supplementary Materials).

### 3.3. Multi-Variable Relationship between MD and Indices of Physical Function

In a linear regression model there was no significant association between MD adherence score as a continuous variable and the following physical function indices: grip strength, Berg Balance Test, 6-min walk, and 10-m walk. Based on age distribution and geriatric considerations, age was grouped into ≤75 years and >75 years and a GLM model was fitted that included adherence to MD, age (grouped), MD by age interaction, gender, BMI, and physical activity.

**Figure 1:**
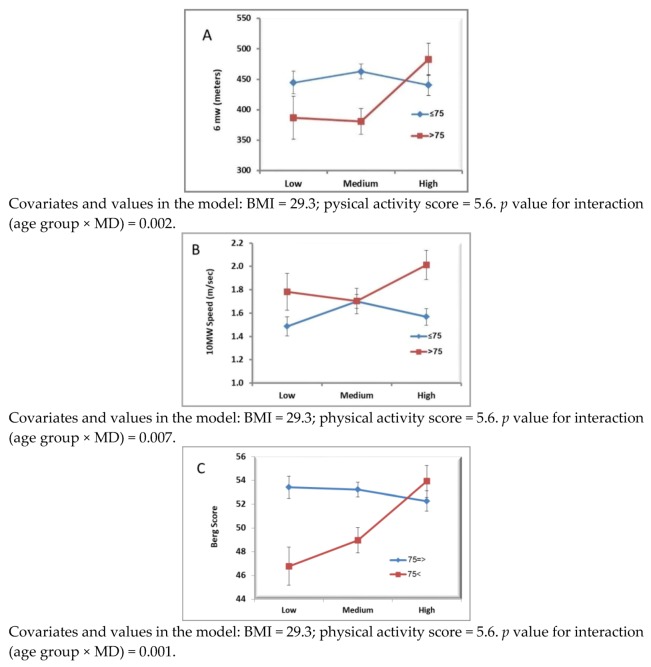
(**A**) Six-min walk test according to MD adherence by age group, controlled for covariates *; (**B**) 10-m walk test according to MD adherence by age group, controlled for covariates *; (**C**) Berg scale according to MD adherence by age group, controlled for covariates *; * Covariates are: gender, BMI, and physical activity.

The age interaction of the multi-variable relationship between MD and indices of physical function is presented in Figure 1. Using GLM, controlling for potential confounders (gender, BMI, and physical activity level), a significant age by MD interaction was identified: a higher adherence to MD (3rd tertile) was associated with longer distance achieved in the 6-min walk test in the participants aged >75 years (Figure 1A), whereas this association was not found for participants aged <75. Similar results were obtained for the 10-m walk test and Berg test: age by MD interaction was significant, indicating that higher speed of walking was associated with MD only in the older age (>75 years) (Figure 1B), and also higher Berg score was associated with higher adherence to MD only in the older age (Figure 1C). Table 3 shows parameter estimates of the full model for the three outcomes in the two age groups.

## 4. Discussion

This cross-sectional analysis of 117 individuals with diabetes over the age of 60 demonstrated that those with higher adherence to the MD were at lower risk for falls and had greater muscle strength as measured by grip strength. Interestingly, these associations did not persist in a linear model and after adjustment for covariate factors. The relationship between physical performance measured by walking speed and walking distance and MD differed by age. Among participants over 75 years, after adjustment for gender, BMI, and physical activity, adherence to MD was associated with a longer distance achieved in the 6-min walk test and higher walking speed in the 10-m walk test.

The importance of these findings is further highlighted by studies that have demonstrated that walking speed is a predictor of mobility limitation and mortality. Thus, a meta-analysis of 34,485 participants demonstrated that a decline in walking speed in the elderly is a significant predictor for mortality [38,39].

The association between MD and walking speed as well as other mobility performance tests has been previously reported among older people in various age groups [13,14,19]. In most studies age was considered as an independent predictor for functional performance and thus was adjusted in the final models. The impact of interaction with age was not presented as far as our review of the literature has found.

Both age [38,39] and diabetes are risk factors for decreased mobility and functional performance. Indeed, older people with diabetes have a 50–80% greater risk for physical disability compared to those without diabetes [40,41]. In a systematic review of 26 studies, diabetes was shown to increase the risk of mobility disability (OR 1.71, 95% CI 1.53–1.91), IADL disability (1.65, 1.55–1.74), and ADL disability (1.82, 1.63–2.04) [42]. In the nationally representative sample of the Mexican Health and Aging Study the rate of disability increased with age. This decline was accelerated among diabetic patients [43].

MD is characterized by a high intake of vegetables, fruits, legumes, and cereals; a moderate to high intake of fish; moderate to low consumption of poultry, meat, and dairy and monounsaturated fatty acids (mainly from olive oil); and a moderate intake of wine. The MD score we have used was validated among elderly [19]. There may be several explanations for the positive association found between this type of diet and physical indices in older people with diabetes. Adherence to MD may be a marker of general healthy behavior patterns; thus, for example, individuals who have high adherence to the MD diet may also engage in more physical activity and therefore have greater grip strength and better walking abilities. It is also possible that the association observed is due to a causal relationship. This may be through anti-oxidant, anti-inflammatory nutrients that comprise the MD and may be increasingly relevant when dealing with diabetes, a disease with higher oxidative stress and inflammation [44,45,46].

The interaction of MD with age may be explained in several ways, one is a selection bias whereby the older cohort may include healthier participants in whom MD may have a stronger contribution. The other is that the age-dependent lower physical function within this well-treated population manifests only in older ages (75 and older). Our findings suggest that high adherence to MD may attenuate this age-dependent decline.

This study has several limitations. First, this is a cross-sectional study, thus the results need to be interpreted with caution and temporality cannot be determined. Second, a limited number of possible confounding variables could be taken into account; this was done using the standardized score approach (taking into account age and gender, BMI, and physical activity). Due to the relatively small sample size additional adjustments could not be performed. However, further support to the findings’ consistency is the repeatability of the findings for 6 MW, 10 MW, and the Berg score. Third, this study pertains to a specific population; study participants were individuals over the age of 60 with relatively long-standing diabetes and high educational attainment (mean of 15.6 years); thus it is not clear if our results may be applicable to other older people with diabetes.

## 5. Conclusions

This study demonstrates an association between the balance indices, risk for falls, muscle strength, aerobic capacity, and MD among older people with diabetes. Due to cross-sectional design, future studies may focus on the longitudinal relationship between MD and these physical indices and provide baseline data for intervention studies aimed at alleviating frailty in this high-risk population.

## Figures and Tables

**Table 1:** Demographic and Medical characteristics according to adherence to MD category.

Variable *	All	Low Adherence (0–2)	Medium Adherence (3–4)	High Adherence (5–9)	*p* for Comparison
Female	46 (39.3)	10 (41.7)	27 (43.5)	9 (29)	0.3877
Age, Mean ± SD	70.6 ± 6.5	70.7 ± 7.4	69.7 ± 4	72.1 ± 5.8	0.2573
Age ≤ 75 years,	87 (74.4)	18 (15.4)	47 (40.2)	22 (18.8)	0.878
Married	89 (76.1)	20 (83.3)	45 (72.6)	24 (77.4)	0.8490
Israel country of birth,	83 (70.9)	17 (70.8)	43 (69.4)	23 (74.2)	0.5166
Lives alone,	17 (14.7)	3 (12.5)	11 (17.7)	3 (10)	0.2009
Education (year)	15.6 ± 2.7	15.3 ± 2.6	15.4 ± 2.7	16.1 ± 2.6	0.3848
Medical characteristic					
Dyslipidemia	114 (97.4)	24 (100.0)	59 (95.2)	31 (100.0)	0.2552
HTN ^1^	100 (85.5)	22 (91.7)	50 (80.6)	28 (90.3)	0.2876
Smoker	3 (2.6)	0 (0)	2 (3.3)	1 (3.2)	0.6691
BMI ^2^	29.24 ± 4.79	29.96 ± 4.62	29.65 ± 5.12	28.19 ± 4.15	0.3010
Diabetes duration (year)	16.93 ± 10.33	19.46 ± 11.13	17.28 ± 10.56	14.87 ± 9.09	0.2609
HbA1c ^3^	7.38 ± 1.13	7.59 ± 1.19	7.32 ± 1.21	7.35 ± 0.82	0.7003
Insulin user	39 (33.6)	9 (37.5)	22 (36.1)	8 (25.8)	0.5562
Diabetes Complication					
Neuropathy	85 (72.6)	17 (70.8)	48 (77.4)	20 (64.5)	0.4103
Retinopathy	22 (19.8)	7 (30.4)	10 (17.5)	5 (16.1)	0.3530
Nephropathy	22(20.8)	6 (27.3)	11 (19.3)	5 (18.5)	0.6963
IHD ^4^	40 (34.5)	9 (37.5)	18 (29.5)	13 (41.9)	0.4660
CVD ^5^	17 (14.7)	3 (12.5)	10 (16.4)	4 (12.9)	0.8553
PVD ^6^	20 (17.1)	7 (29.2)	10 (16.1)	3 (9.7)	0.1563

* Data are shown as *N* (%) for counts and Mean ± SD for continuous data. SD: Standard Deviation. ^1^ HTN: Hypertension. ^2^ BMI: Body Mass Index. ^3^ HbA1c: Hemoglobin A1c (Glycated hemoglobin). ^4^ IHD: Ischemic heart disease. ^5^ CVD: Cardiovascular disease. ^6^ PVD: Peripheral vascular disease.

**Table 2:** Physical and aerobic strength and balance according to adherence to the Mediterranean Diet category.

Variable	All	Low Adherence (0–2)	Medium Adherence (3–4)	High Adherence (5–9)	*p* for Comparison
Pre-frail	13 (11.1)	5 (20.8)	6 (9.7)	2 (6.5)	0.2115
Frail	3(2.6)	1 (4.2)	2(3.2)	0 (0.0)	0.5568
Grip strength ^1^	−0.53(0.88)	−0.93 (0.82)	−0.51 (0.89)	−0.29 (0.84)	0.0263
Balance ^2^	52.59 ± 5.02	51.25 ± 6.35	52.53 ± 4.96	53.52 ± 3.86	0.2568
Balance category ^2^					
Increased risk of falls	2 (1.7)	0 (0.0)	2 (3.2)	0 (0.0)	0.0246
Needs a walking aid for safe walking	11 (9.4)	6 (25.0)	2 (3.2)	3 (9.7)	
No increased risk of falls	104 (88.9)	18 (75.0)	58 (93.5)	28 (90.3)	
FR ^3^	29.7 ± 6.3	27.7 ± 6.5	29.8 ± 6.4	31.1 ± 5.9	0.1716
FSST ^4^	12.08 ± 4.57	13.34 ± 6.83	12.22 ± 4.22	11.15 ± 3.15	0.2576
6-min walk ^5^	458.4 ± 114.0	409.8 ± 143.5	465.6 ± 107.3	477.4 ± 95.4	0.0658
30-s chair stand	11.6 ± 4.3	10.6 ± 4.9	11.9 ± 4.3	11.8 ± 3.7	0.4612
Timed-Get-Up-and-Go	7.59 ± 3.65	8.31 ± 3.20	7.78 ± 4.40	6.73 ± 1.90	0.2512
10-min walk (seconds)	6.77 ± 5.67	7.49 ± 3.61	6.98 ± 7.47	5.90 ± 1.34	0.5615
10-min walk (speed m-s)	1.70 ± 0.45	1.54 ± 0.51	1.73 ± 0.46	1.77 ± 0.37	0.1322

Data are shown *N* (%) for counts and Mean ± SD for continuous data. ^1^ Grip strength was assessed using the Jammer dynamometer; presented are *z*-scores of the sex standardized scores. ^2^ Balance was assessed using the Berg Balance Scale (BBS). Scores below 36 indicate an increased risk for falls; scores between 37 and 45 indicate need of a walking aid in order to walk in a safe manner; >45 no increased risk of falls (independent walker). ^3^ Functional Reach (FR), a sub-test of the BERG Balance Scale, presented as distance in cm. ^4^ Four Square Step Test (FSST) presented as time in seconds to complete the task. ^5^ Presented are *z*-scores of the age and sex standardized scores in meters.

**Table 3:** GLM ^§^ models for 6 mw, 10 mwt, and Berg scale across age categories.

Age Group	≤75 (*n* = 87)						>75 (*n* = 30)					
Parameter	6 MWT (m)		10 MWT Speed (m/s)		Berg Scale		6 MWT (m)		10 MWT Speed (m/s)			
	B ± Std.E	*p*	B ± Std.E	*p*	B ± Std.E	*p*	B ± Std.E	*p*	B ± Std.E	*p*	B ± Std.E	*p*
Gender (male)	44.85 ± 18.64	0.016	0.26 ± 0.08	0.001	0.94 ± 0.98	0.334	97.11 ± 25.42	0.000	0.4 ± 0.11	0.000	2.63 ± 1.22	0.031
BMI *	−8.42 ± 1.77	0.000	−0.03 ± 0.01	0.000	−0.26 ± 0.09	0.004	−4.43 ± 4.04	0.273	0 ± 0.02	0.808	0.07 ± 0.19	0.725
MD ** (low vs. high)	−20.1 ± 25.17	0.425	−0.11 ± 0.11	0.305	0.56 ± 1.32	0.671	−123.53 ± 37.26	0.001	−0.36 ± 0.16	0.019	−7.88 ± 1.79	<0.001
MD (medium vs. high)	24.12 ± 20.65	0.243	0.09 ± 0.09	0.297	0.6 ± 1.08	0.577	−103.24 ± 26.17	<0.001	−0.39 ± 0.11	<0.001	−5.01 ± 1.25	<0.001
Age	−7.78 ± 2.11	0.000	−0.04 ± 0.01	0.000	−0.14 ± 0.11	0.216	0.24 ± 4.44	0.956	0 ± 0.02	0.827	0.18 ± 0.21	0.394
Physical activity score	16.68 ± 4.64	0.000	0.05 ± 0.02	0.012	0.67 ± 0.24	0.006	36.6 ± 6.78	0.000	0.12 ± 0.03	0.000	1.94 ± 0.32	<0.001

* BMI—Body Mass Index; ** MD—Mediterranean Diet score. Corrected for Gender, BMI, age, and physical activity score; ^§^ Generalized Linear Model.

## Supplementary Materials

The following are available online at http://www.mdpi.com/2072-6643/10/6/767/s1, File S1: Measurement of physical indices; Table S1: Nutrients intake across levels of MD adherence.
