# Supplementary material for: The Association between Mediterranean Diet and the Risk of Falls and Physical Function Indices in Older Type 2 Diabetic People Varies by Age
Source: Nutrients. 2018 Jun 14;10(6):767. doi: 10.3390/nu10060767 (PMC6024581; doi:10.3390/nu10060767)
Supplement: Supplementary file 1 [file nutrients-10-00767-s001.pdf]

Supplementary

File S1. Measurement of physical indices.

Gait, Balance, & Aerobic Capacity Measurement

The following instruments were used to assess gait speed, balance, and aerobic capacity by the physiotherapist.

Timed-Get-Up-and-Go [1-4]

The objective of this test is to measure the ability of a person to: stand up, walk, turn around, and sit down safely in a timely manner. The test examines most mobility skills. The participant is told to get up from a chair with handles, walk 3 meters, turn, walk back, and sit down again. The score is according to the length of time in seconds to complete the task. The score is categorized according to the risk for falls and independent walking. The following cut-offs are conventionally used: less than 14 seconds = independent mobility; 15–20 seconds = semi-independent mobility, may have a somewhat increased risk for falls & needs further evaluation, some may need a walking aid; 20–30 seconds = dependent mobility: need help walking, 50% with a cane, 40% walker, 10% supervision. Some will need help in transfers, and most will require help using the toilet. Many in this category won't go outside the home alone.

Data suggests that the Timed-Get-Up-and-Go" test is a reliable and valid test for quantifying functional mobility that may also be useful in following clinical change over time.

6-m.inute walk [1,5-7]

The six-minute walk test (6MWT) measures the distance an individual is able to walk during a total of six minutes on a hard, flat surface. The goal is for the individual to walk as far as possible in six minutes. The individual is allowed to self-pace and rest as needed as they traverse back and forth along a marked walkway. The six-minute walk distance in healthy adults has been reported to range from 400 to 700 m. People with lower vs. higher scores on the 6-minute walk are at higher risk for falls, disability, frailty, hospitalization, and death.

10-.meter walk [8]

The test examines the pace and number of steps it takes a person to pass 10 meters. A route of 10 meters is marked by two lines and a chair is placed two meters past the runway end line.

The subject starts the test two meters before the runway and goes 14 meters (two meters for acceleration at the beginning and two meters for deceleration at the end). The score achieved is determined by the time elapsed by the participant during walking along the middle 10 meters.

The subject performs the test four times, the first two times are for practice: measurement occurs only during the third and fourth times. In addition to measuring the speed, the number of steps required to cross the short distance are also counted. Studies have a shown that better gait speed is associated with a lower risk for functional decline, hospitalization, and mortality.[9,10]

Berg Balance Scale

The Berg Balance Test includes 14 tasks which evaluate static and dynamic balance. Each task receives a score of 0 to 4 points - depending on the quality and task execution time.[40-42] The maximum score is 56 points. The scores are classified in the following manner:

1. Scores below 36 indicate impairment with an increased risk for falls.
2. Scores between 37 and 45 indicate need for a walking aid in order to walk in a safe manner.
3. Scores above 45 indicate an independent walker without an increased risk of falls.

The equipment used for the Berg Balance Test is a step stool, a mat table, a chair with arms, a tape measure, a stopwatch, a pen, and a table. Studies have shown that individuals with scores indicating impaired balance are at increased risk of falls resulting in hospitalizations and deaths.[11]

Four Square Step Test [12]

The test evaluates dynamic balance in a high functional level_and features walk forward, backwards, left, and right above two 90 cm and 2.5 cm high long sticks that divide the floor into four squares. The participant stands in square 1 facing square 2. The goal is to walk as quickly as possible through all the squares in the following order: from 1 to 2,3,4,1,4,3,2, and 1 without touching the sticks. The score is the time required to complete the entire route.

1. Donovan K, Lord SE, McNaughton HK, Weatherall M. Mobility beyond the clinic: the effect of environment on gait and its measurement in community-ambulant stroke survivors. *ClinRehabil* 2008/6; **22**(6): 556–63.
2. van Iersel MB, Hoefsloot W, Munneke M, Bloem BR, Olde Rikkert MG. Systematic review of quantitative clinical gait analysis in patients with dementia. *ZGerontolGeriatr* 2004/2; **37**(1):27–32.
3. van Iersel MB, Munneke M, Esselink RA, Benraad CE, Olde Rikkert MG. Gait velocity and the Timed-Up-and-Go test were sensitive to changes in mobility in frail elderly patients. *J Clin Epidemiol* 2008; **61**(2): 186–91.
4. van Iersel MB, Ribbers H, Munneke M, Borm GF, Rikkert MG. The effect of cognitive dual tasks on balance during walking in physically fit elderly people. *Arch Phys Med Rehabil* 2007; **88**(2): 187–91.
5. Bellet RN, Adams L, Morris NR. The 6-minute walk test in outpatient cardiac rehabilitation: validity, reliability and responsiveness--a systematic review. *Physiotherapy* 2012; **98**(4): 277–86.
6. Larsson UE, Reynisdottir S. The six-minute walk test in outpatients with obesity: reproducibility and known group validity. *Physiother Res Int* 2008; **13**(2): 84–93.
7. Hamilton DM, Haennel RG. Validity and reliability of the 6-minute walk test in a cardiac rehabilitation population. *J Cardiopulm Rehabil* 2000; **20**(3): 156–64.
8. Rossier P, Wade DT. Validity and reliability comparison of 4 mobility measures in patients presenting with neurologic impairment. *ArchPhysMedRehabil* 2001/1; **82**(1): 9–13.
9. Studenski S, Perera S, Patel K, et al. Gait speed and survival in older adults. *JAMA* 2011; **305**(1): 50–8.
10. Fritz S, Lusardi M. White paper: "walking speed: the sixth vital sign". *J Geriatr Phys Ther* 2009; **32**(2): 46–9.
11. Berg KO, Wood-Dauphinee SL, Williams JI, Maki B. Measuring balance in the elderly: validation of an instrument. *CanJPublic Health* 1992/7; **83 Suppl 2**: S7–11.
12. Dite W, Temple VA. A clinical test of stepping and change of direction to identify multiple falling older adults. *Arch Phys Med Rehabil* 2002; **83**(11): 1566–71.

Table S1. Nutrients intake across levels of MD adherence.

Nutrient	Low Adherence (0-2)	Medium Adherence (3-4)	High Adherence (5-9)	p for comparison
Total energy(Kcal)	2040.99 (516.94)	2426.91 (718.90)	2587.02 (776.33)	0.016
Protein (g)	105.03 (26.50)	115.37 (37.32)	116.34 (29.53)	0.378
Total lipids (g)	84.56 (31.81)	94.30 (29.15)	101.00 (25.00)	0.113
Carbohydrate (g)	223.25 (78.94)	290.14 (108.77)	312.95 (128.17)	0.009
% protein/energy	21.10 (4.89)	19.26 (3.59)	18.14 (2.82)	0.016
% carb/energy	43.82 (11.53)	47.37 (7.45)	47.42 (6.78)	0.176
% fat/energy	37.00 (9.00)	35.27 (5.61)	35.83 (4.94)	0.52
Total dietary Fibers (g)	28.54 (7.96)	37.66 (11.11)	42.07 (11.75)	<0.001
Calcium (mg)	923.51 (348.36)	1020.79 (431.82)	944.26 (265.54)	0.466
Iron (mg)	13.15 (4.43)	15.42 (5.08)	17.41 (6.05)	0.013
Magnesium (mg)	361.95 (87.73)	456.38 (127.24)	493.85 (137.58)	0.001
Phosphorus (mg)	1674.26 (454.93)	1921.54 (666.05)	1878.69 (454.41)	0.204
Potassium (mg)	3590.75 (780.62)	4465.13 (1250.66)	4670.87 (1029.77)	0.001
Sodium (mg)	3786.51 (833.38)	4578.89 (1376.57)	4786.21 (1192.69)	0.009
Zinc (mg)	13.15 (4.51)	14.23 (4.66)	14.57 (3.63)	0.467
Selenium (mcg)	130.24 (30.53)	158.85 (54.77)	161.39 (54.19)	0.042
Vitamin A RAE(mcg)	1043.61 (396.00)	1152.15 (499.62)	1227.85 (619.44)	0.424
Vitamin E (alpha tocopherol) (mg)	9.45 (3.13)	11.29 (3.40)	12.74 (3.29)	0.002
Vitamin D(IU)	109.78 (73.10)	105.09 (76.89)	90.09 (50.33)	0.521
Vitamin C (mg)	165.79 (72.55)	212.77 (102.87)	276.94 (134.93)	0.001
Vitamin K (mcg)	181.58 (91.70)	228.62 (87.14)	288.27 (113.81)	<0.001
Thiamin (mg)	1.58 (0.63)	1.87 (0.63)	2.01 (0.66)	0.044
Riboflavin (mg)	2.47 (0.85)	2.62 (0.97)	2.50 (0.74)	0.699
Niacin (mg)	24.49 (7.58)	28.03 (9.68)	28.21 (8.86)	0.227
Pantothenic acid (mg)	6.74 (1.55)	7.74 (2.38)	7.94 (2.33)	0.106
Vitamin B6 (mg)	2.43 (0.86)	2.86 (1.09)	2.91 (0.83)	0.137
Folate (mcg)	283.54 (76.90)	358.08 (107.38)	421.07 (133.86)	<0.001
Vitamin B12 (mcg)	6.06 (3.17)	5.82 (3.15)	5.85 (4.29)	0.958
Fatty acids - total trans (g)	1.11 (0.54)	1.63 (0.80)	1.74 (0.64)	0.004
Fatty acids - total saturated (g)	25.85 (11.97)	28.63 (10.32)	28.76 (8.53)	0.487

Fatty acids - total monounsaturated (g)	30.20 (11.31)	34.32 (10.63)	37.32 (9.42)	0.048
Fatty acids - total polyunsaturated (g)	21.81 (11.64)	24.87 (8.28)	28.24 (7.55)	0.03
18:1 Oleic acid (g)	27.32 (9.87)	31.42 (9.92)	34.05 (8.70)	0.039
18:3 alpha linoleic acid (n-3) (g)	1.96 (0.84)	2.18 (0.65)	2.41 (0.54)	0.044
20:5 EPA (g)	0.03 (0.02)	0.03 (0.02)	0.04 (0.02)	0.05
22:6 DHA (g)	0.15 (0.11)	0.18 (0.13)	0.23 (0.13)	0.087

Data are shown as Mean (SD).

RAE- Retinol Activity Equivalents.

% protein/energy=protein (g)X4/total energy (kcal).

% carb/energy= carbohydrates (g) X4/ total energy (Kcal).

% fat/energy= fat (g)×9/ total energy (Kcal).

EPA - Eicosapentaenoic acid.

DHA - Docosahexaenoic acid.
